# Translation of liver stage activity of M5717, a *Plasmodium* elongation factor 2 inhibitor: from bench to bedside

**DOI:** 10.1186/s12936-022-04171-0

**Published:** 2022-05-15

**Authors:** Akash Khandelwal, Francisca Arez, Paula M. Alves, Lassina Badolo, Catarina Brito, Christoph Fischli, Diana Fontinha, Claude Oeuvray, Miguel Prudêncio, Matthias Rottmann, Justin Wilkins, Özkan Yalkinoglu, Wilhelmina M. Bagchus, Thomas Spangenberg

**Affiliations:** 1 The healthcare business of Merck KGaA, Darmstadt, Germany; 2 iBET, Instituto de Biologia Experimental e Tecnológica, Oeiras, Portugal; 3 Instituto de Tecnologia Química e Biológica António Xavier, Universidade Nova de Lisboa, Oeiras, Portugal; 4 Department of Medical Parasitology and Infection Biology, Swiss Tropical and Public Health Institute, Basel, Switzerland; 5 University of Basel, Basel, Switzerland; 6 Faculdade de Medicina, Instituto de Medicina Molecular João Lobo Antunes, Universidade de Lisboa, Lisboa, Portugal; 7 Global Health Institute of Merck, Ares Trading S.A (a subsidiary of Merck KGaA, Darmstadt, Germany), Eysins, Switzerland; 8 Occams, Amstelveen, The Netherlands; 9 Merck Institute for Pharmacometrics, MerckSerono S.A. (an affiliate of Merck KGaA Darmstadt, Germany), Lausanne, Switzerland

**Keywords:** M5717, *Plasmodium* elongation factor 2, Hepatic, Spheroids, 3R, Modelling, Population Pharmacokinetics

## Abstract

**Background:**

Targeting the asymptomatic liver stage of *Plasmodium* infection through chemoprevention could become a key intervention to reduce malaria-associated incidence and mortality.

**Methods:**

M5717, a *Plasmodium* elongation factor 2 inhibitor, was assessed in vitro and in vivo with readily accessible *Plasmodium berghei* parasites. In an animal refinement, reduction, replacement approach, the in vitro IC_99_ value was used to feed a Population Pharmacokinetics modelling and simulation approach to determine meaningful effective doses for a subsequent *Plasmodium* sporozoite-induced volunteer infection study.

**Results:**

Doses of 100 and 200 mg would provide exposures exceeding IC_99_ in 96 and 100% of the simulated population, respectively.

**Conclusions:**

This approach has the potential to accelerate the search for new anti-malarials, to reduce the number of healthy volunteers needed in a clinical study and decrease and refine the animal use in the preclinical phase.

**Graphical Abstract:**

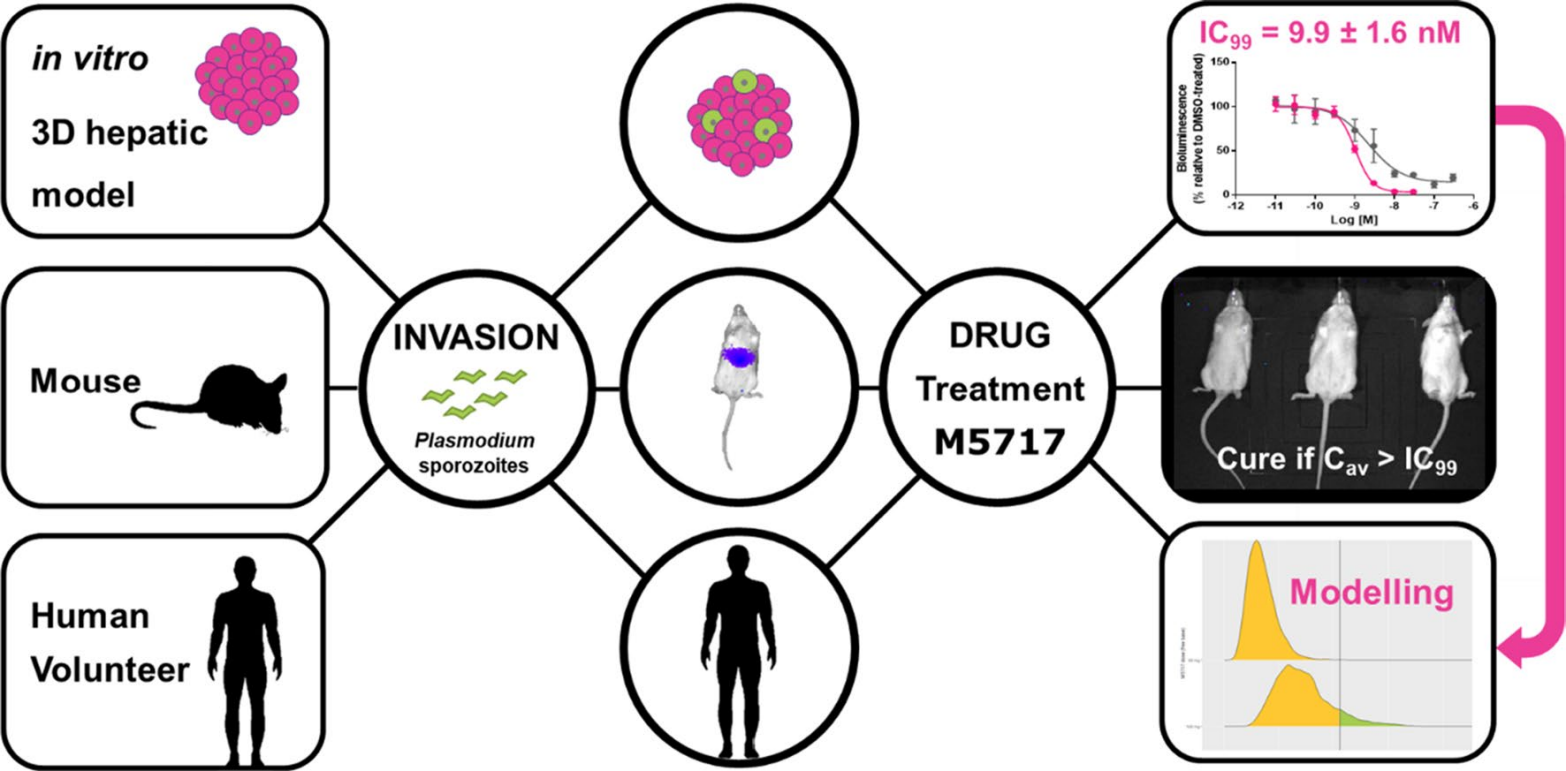

## Background

Despite the availability of various treatments for malaria, morbidity and mortality associated with this disease remain high [1]. The aim of the World Health Organization (WHO) is to reduce mortality, incidence and re-establishment of malaria by 90% by 2030 [1].

Malaria is caused by protozoan parasites of the genus *Plasmodium*. The initial site of mammalian infection following a mosquito bite is the liver in which sporozoites (spz) mature into merozoites that are eventually released into the bloodstream to invade erythrocytes leading to the pathology [2]. Consequently, targeting the asymptomatic liver stage of infection through chemoprevention could become a key intervention to prevent symptomatic malaria and achieve the WHO objective [3].

Model informed dose selection to initiate early phase of clinical development has become a powerful tool. In malaria, volunteer infection studies (VIS) are widely used for drug and vaccine development to achieve early proof-of-principle [4]. For the liver stage of *Plasmodium* infection, the generation of data to feed Population Pharmacokinetics (PopPK) models to set the clinical starting dose essentially relies on in vivo mouse models, due to limited availability of human-based in vitro models [5–7].

Here, a first attempt was made using a recently developed in vitro 3D infection platform that employs hepatic cell line-derived spheroids to generate an effective average concentration (C_av_) of M5717, a new anti-malarial that acts as a *Plasmodium* elongation factor 2 inhibitor, which is also active against the liver stage of infection. The in vitro derived values were set as a target concentration to prevent liver stage infection. Complementarily, those concentrations were employed in an in vivo model to assess the ability of M5717 to prevent the appearance of a blood stage infection in mice challenged with *Plasmodium* spz. A previously developed PopPK model based on clinical Phase 1 data was used to simulate doses that would exceed the target in vitro effective concentration [4, 8, 9]. M5717 has entered a liver stage *Plasmodium* spz induced VIS (NCT04250363) in 2021 employing the predicted doses described in this article [8, 10].

## Methods

### In vitro hepatic *Plasmodium* infection

HepG2 cells (ATCC) were cultured in an incubator with humidified environment at 37 °C with 5% CO_2_. Cell expansion was performed in 2D static culture, in low glucose DMEM (Thermo Fisher Scientific) supplemented with 1% (v/v) penicillin/streptomycin and 10% (v/v) FBS. Cells were passaged twice a week, at 5 × 10^4^ cell/cm^2^. For 3D culture, cells were suspended in the same culture medium, supplemented with 10% filtered FBS (v/v) and inoculated as single cell suspensions (3 × 10^5^ cell/mL) in 125 mL (from Corning, Merck KGaA). After aggregation medium exchanges were performed with 5% filtered FBS culture medium. Agitation was induced by magnetic stirring and adjusted to promote spheroid formation and sustain their long-term culture (up to 30 days), as described before [9].

Infection of HepG2 spheroids in dynamic conditions was performed in 30 mL spinners (from ABLE Biott Corporation), at a cell concentration of 5 × 10^5^ cell/mL and a cell/spz ratio of 1:1. For infection in static conditions, spheroids were transferred to 96-well plates (2.5 × 10^4^ cell/well) and exposed to *P. berghei-Luc* in a 1:2 cell/spz ratio; Cultures were challenged with the drugs during the development phase of the parasite, from 24 h up to 48 h post-infection (pi). For dose-dependence assays, infection rate and metabolic activity were assessed at 48 h pi, whereas for the time-dependence assays, infection rate and dsDNA concentration were assessed up to 84 h pi. *P. berghei*-luc was detected by bioluminescence, with the Firefly Luciferase Assay Kit (Biotium), following the manufacturer’s instructions, as previously described [9]. The normalized data were fitted to dose-response curves by nonlinear regression analysis and IC_50_ values determined using GraphPad Prism version 6 for Windows (GraphPad Software). The lowest drug concentration that inhibited infection by 99% of the DMSO-treated controls was considered the minimal inhibitory concentration or IC_99_.

### Liver stage *Plasmodium* infection in vivo

Naïve female NMRI mice were infected with 1 × 10^5^ spz of *P. berghei* mCherry ANKA-Luci-GFP by intravenous (i.v.) injection in the tail vein. At + 24 h pi, single oral (p.o.) dose drug treatment with M5717 (0.3, 0.6, 1.5, 3 and 30 mg/kg) was administered. Compounds were dissolved in a vehicle of 7% Tween 80 and 3% ethanol. *Plasmodium* liver infection was assessed in vivo at 23 and 48 h pi in anesthetized animals employing an IVIS Lumina II system after following i.v. injection of 5 mL/kg D-Luciferin (30 mg/mL). Blood-stage parasitaemia was measured 7, 14, 17, 21, 24, 28, 31 and 35 days pi by light microscopy on Giemsa-stained blood smears, and blood-stage positive mice were euthanized. Mice without detectable parasitaemia until day 35 pi (33 days post treatment) were considered as cured and were euthanized. Blood samples were collected to assess the drug exposure level to determine the C_av24h_. One mouse received 10 mg/kg atovaquone (ATO) as a positive control and three mice served as untreated controls.

#### PopPK modelling

PopPK modelling was performed with the nonlinear mixed effect modelling software NONMEM (version 7.4.3, ICON Development Solutions, Dublin, Ireland) using the first order conditional estimation method with interaction (FOCEI) supplemented with Perl-speaks-NONMEM (PsN) (version 4.9.0) [8, 11]. Microsoft R Open (version 3.5.1, Microsoft, Redmond, Virginia, USA) was used for general scripting, data management, goodness of fit analyses and model evaluation.

#### Dose simulations for prevention of the development of blood-stage parasitaemia

The final PopPK model was used for simulations to identify doses for prophylaxis in R using mrgsolve [8]. Using the variance-covariance matrix obtained from NONMEM, 1000 sets of parameters were generated. For each set, 1000 sets of individuals were generated using between-subject variability estimates. From this pool, 10,000 individuals were randomly sampled without replacement and used for simulations. Doses were tested as combinations of 100 and 30 mg of M5717 free base as these were the available capsule strengths of the formulations.

## Results

### Liver stage *Plasmodium* infection in vitro

**Fig. 1:**
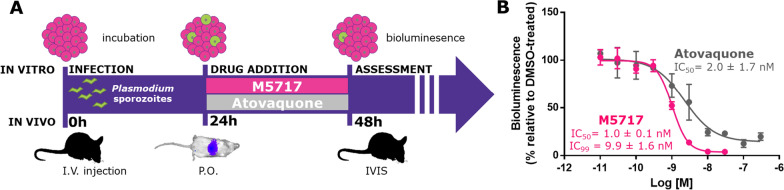
**A** Scheme of infection, treatment and bioluminescent readouts for HepG2 cell spheroids and mice infected with *P. berghei*-Luc. **B** Dose−response curves and IC_50_ determinations of M5717 (pink) and atovaquone (gray; positive control) in HepG2 spheroids infected by *P. berghei*-Luc under static conditions at 2.5 × 10^4^ cell/well in a 1:2 cell:spz ratio. The dots and error bar represents mean and standard deviation from three independent experiments

M5717 showed hepatic anti-plasmodial activity when incubated with luciferase-expressing *Plasmodium berghei*-Luc-infected HepG2 spheroids from 24 to 48 h post-infection (pi) [9]. The drug showed remarkable potency against *P. berghei*-Luc-infected HepG2 spheroids, with an IC_50_ of 1.0 ± 0.1 nM and an IC_99_ of 9.9 ± 1.6 nM (Fig. 1) [10]. Therefore, the latter concentration, effective in low-metabolizing HepG2 hepatic spheroids, was used for translation to liver stage activity in vivo. Given the large excess of FBS-derived proteins present in the in vitro medium compared with the drug concentration, only the human blood-to-plasma ratio (B/P = 1.6) was used to convert the IC_99_, an effective average concentration over 24 h, to the corresponding area under the curve (AUC) drug exposure of 180 ng∙h/mL in human plasma.

### Liver stage *Plasmodium* infection in vivo

The *P. berghei*-Luc liver stage mouse model was used to validate the IC_99_ value obtained in vitro [9]. Our results show that the 30, 3 and 1.5 mg/kg single oral doses of M5717 were fully effective in preventing the appearance of blood stage infection in all treated mice, while 0.3 mg/kg and 0.6 mg/kg did not, or only partially prevented the development of parasitaemia (Table 1).

**Table 1:** In vivo average blood concentration of M5717 over 24 h

Dose in mg/kg	Cure^a^	Mean whole blood concentration in ng/mL at 2 h (n = 3)	SD	Mean whole blood Concentration in ng/mL at 24 h (n = 3)	SD	AUC_0 − 24 h_ in ng/mL.h (n = 3)	Cav_0 − 24 h_^b^ in nM (n = 3)	Cav_0 − 24 h_^b^/IC_99_ ratio
0.3	0/5^c^	7.36	0.63	4.91	0.55	142.33	3.6	0.4
0.6	1/2	–	–	–	–	–	–	~ 1^d^
1.5	2/2	–	–	–	–	–	–	~ 2^d^
3	3/3	84.83	25.03	41.97	9.73	1479.63	37.9	4
30	3/3	1058	301.48	576.3	169.01	19035.3	487.1	49

^a^Based on a > 30-day follow-up of blood stage parasitaemia. Atovaquone (10 mg/kg) was used as a positive control in these experiments

^b^Correction was made for Blood/Plasma ratio (mouse B/P = 3.52) as only the plasma concentrations are able to interact with the parasites. No plasma protein correction was made here as both in vitro and in vivo contained proteins at high levels

^c^Pooled batches

^d^Estimation based on observed dose linearity across doses tested. A noncompartmental analysis was performed to determine the AUC_0−24 h_ using the Phoenix WinNonlin program (version 6.3)

To correlate the in vivo exposures obtained at various oral doses with the effective concentrations previously determined in our in vitro 3D hepatic cell model, the AUC_0−24 h_ was converted to an average plasma concentration of the drug (C_av0−24 h_) over 24 h and was compared to the compound’s IC_99_ in vitro (Table 1). Importantly, both models employed a similar protocol, with a 24 h exposure time, between 24 and 48 h pi (Fig. 1). All mice for which the drug was fully effective in preventing the appearance of blood stage parasites achieved systemic plasma C_av0−24 h_ > 10 nM. With the non-preventive dose of 0.3 mg/kg, a C_av0−24 h_ of 4 nM was observed, which corresponded to only half of the IC_99_ and did not prevent the development of blood-stage parasites. A 0.6 mg/kg dose produced a C_av0−24 h_ close to the IC_99_ value, preventing only partially the development of blood-stage parasitaemia.

Therefore, the use of the in vitro IC_99_ concentration of 10 nM as a relevant parameter for M5717’s efficacy in *P. berghei*-infected liver stages was supported by the average in vivo blood concentration of M5717 over 24 h, as inferred from the mouse model. Consequently, the IC_99_ value was converted into a target exposure value, i.e. AUC_0 − 24 h_ of 180 ng∙h/mL that can be used for a PopPK model developed based on Phase 1 clinical trial exposure data to simulate doses that would exceed the aforementioned AUC value.

$${\text{IC}}_{{99}} = {\text{C}}_{{{\text{av}}24{\text{h}}}} = 10{\text{ nM}};{\text{ MW}} = 462.57{\text{ g}}/{\text{mol}};{\text{ B}}/{\text{P}} = 1.6$$

$${\text{AUC}}_{{0{-}24{\text{h}}}} = (10{\text{nMol}}/{\text{L}}~ \times ~24{\text{ h}}~ \times ~1.6~ \times ~462.57{\text{g/mol}})/1000 = 180{\text{ ng}} \cdot {\text{h}}/{\text{mL}}$$

### PopPK modelling

The model was derived from results obtained from the completed Phase 1 clinical trial [4, 8]. Briefly, M5717 PK was three-compartmental, with first-order elimination, a transit absorption model in combination with first-order absorption, and a recirculation model to account for a secondary peak between 24 and 30 h. Body weight was included a priori on clearance and volume parameters. The model adequately regenerated the data used to create it as evidenced by visual predictive checks; standard diagnostic plots revealed no unacceptable trends.

### Dose simulations for prevention of the development of parasitaemia

**Fig. 2:**
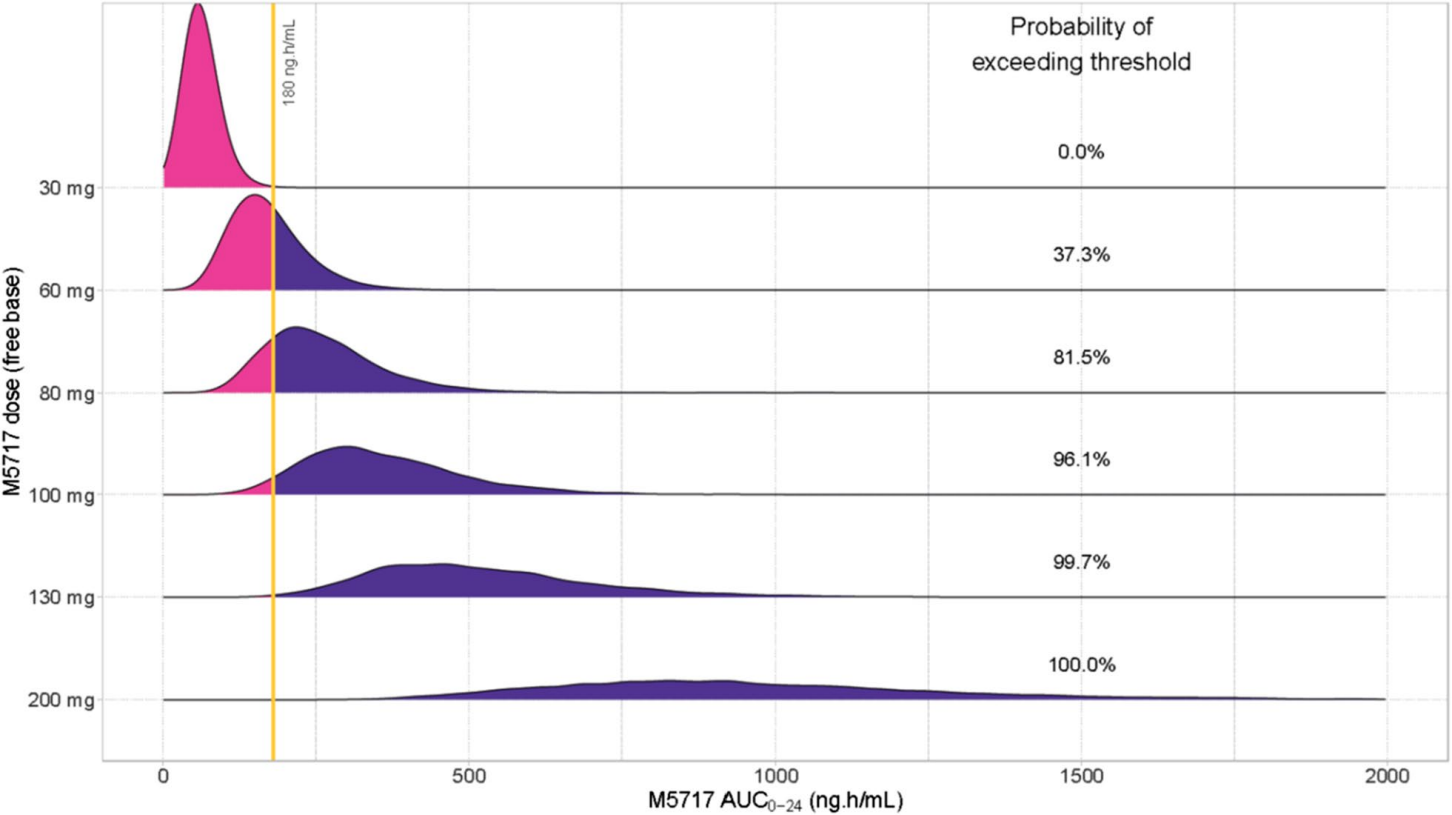
Probability of exceeding (purple) AUC_0 − 24_ of 180 ng∙h/mL (IC_99_) (yellow bar). X axes represents the total dose of M5717 as a free base and the Y axes indicated the simulated M5717 area under the curve

Assuming a human target exposure of AUC_0−24 h_ of 180 ng∙h/mL, simulations for the dosing required to prevent blood stage infection were made assuming (i) full and direct extrapolation of in vitro results to humans, (ii) similar susceptibility between *Plasmodium* species and to M5717 due to high sequence homology (i.e., 98.2% BLAST *P. falciparum* PF3D7_1451100 versus *P. berghei* PBANKA_1314800), (iii) similar relevance of the IC_99_ (C_av_) parameter, and (iv) a blood-to-plasma ratio in humans of 1:6. The results suggested that a 30 mg dose would not provide sufficient exposure, while 80, 100, 130 and 200 mg of M5717 would be required for parasite clearance to be observed in 81.5, 96.1, 99.7 and 100 of subjects, respectively (Fig. 2).

## Discussion

A recently developed in vitro 3D *Plasmodium* infection platform using hepatic cell line-derived spheroids was used as a translational tool for the selection of active molecules against the liver stage of *Plasmodium* infection. Here, the exposure-efficacy relationship obtained from the in vitro model was supported by in vivo data. A starting dose for a chemoprophylactic clinical trial was predicted by combining the in vitro data with PopPK models simulations derived from Phase 1 clinical data.

Using M5717 as an example, a four-step approach to validate the preclinical translatability of the in vitro model was setup by (i) determining the IC_99_ in the in vitro platform over a given period of time (ii) cross validating the C_av_ with the in vivo* P. berghei* model (iii) feeding a PopPK model, and, (iv) defining experimental doses to be evaluated in a spz-induced VIS.

M5717 is well tolerated in humans and exhibits a pharmacokinetic profile characterized by a long half-life (146–193 h at doses ≥ 200 mg). The drug showed potent activity against the hepatic stages of *P. berghei* in both in vitro and in vivo models, after infection has been established indicating that M5717 acts during the parasite’s intrahepatic developmental process. As the emphasis was placed on the patent liver infection, drug treatment only occurred between 24 and 48 h pi with *P. berghei*-Luc spz, to ensure that the drug was present throughout the *P. berghei* liver stage development phase. Upon validation of the relevance of the IC_99_ by comparing with the C_av_ arising from the corresponding in vivo experiments, and assuming a similar susceptibility of *P. berghei* and *P. falciparum* to M5717 due to the high sequence homology of the drug target, the in vitro IC_99_ was used as the target concentration that would lead to prevention of blood stage infection. In both models, the data showed that over a period of 24 h, a C_av0−24 h_ of 10 nM was required for M5717 to completely clear an ongoing *P. berghei*-Luc hepatic infection and could be converted into a target AUC_0−24 h_ of 180 ng∙h/mL.

Assuming that an exposure of 180 ng∙h/mL would be required to achieve full protection, the next step was to use the PopPK model to simulate doses that would exceed the target AUC in majority of the subjects. The data suggested that a dose range of 100–200 mg would be effective for prophylaxis in humans as they exceed the target exposure in 96% and 100% of the simulated population, respectively.

In contrast to blood-stage induced VIS, where 800 mg M5717 led to clearance of the parasitaemia, the simulations based on the current study suggested that doses for prevention may be much lower than curative doses [4].

If the predicted dose and relevant exposure obtained using the 3D hepatic cell platform for *Plasmodium* infection are confirmed in the spz-induced VIS conducted with M5717 (NCT04250363), the translatability of the platform will be strengthened, paving the way for the reduction and refinement of animal use, ultimately potentially replacing in vivo experiments.

Nevertheless, this approach requires careful consideration for other drugs in development as limitations could occur if the anti-plasmodial target displays poor homology between rodent and human parasites. Alternatively, more onerous *P. falciparum*-based models may have to be used. Also, depending on its mode of action, drug exposure times during in vitro experiments may also need to be adjusted. Therefore, the relevance of the IC_99_ or C_av_ parameters may need to be addressed on a case-by-case basis. Availability of a population PK model based on human data is crucial for the simulation of doses for chemoprevention and non-availability of these data and model hinders the application proposed approach to compounds that have not yet entered Phase 1 clinical evaluation.

## Conclusions

For the first time in drug development against the liver stage of infection by malaria parasites, PK and PD data emerging from an in vitro platform combined with a PopPK modelling and simulation were employed to predict the starting dose in humans to be used against the pre-erythrocytic stage of a new anti-malarial compound. The translatability of the in vitro platform has been compared with PK and PD data obtained from in vivo mice experiments and will be further validated when the results of the spz-induced VIS will be available.

Therefore, this non-traditional method has the potential to accelerate the search for new anti-malarials, to reduce the number of healthy volunteers needed in a clinical study and decrease and refine the animal use in the preclinical phase, thus aligning research to the National Centre for the 3Rs principle (NC3R) [7].

## Data Availability

All individual datasets are available.
